# Putative Pathogenic Genes of *Leptospira interrogans* and *Leptospira weilii* Isolated from Patients with Acute Febrile Illness

**DOI:** 10.3390/tropicalmed7100284

**Published:** 2022-10-05

**Authors:** Amira Wahida Mohamad Safiee, Mohammad Ridhuan Mohd Ali, Muhammad Zarul Hanifah Md Zoqratt, Tan Hock Siew, Chua Wei Chuan, Lee Lih Huey, Mohd Hashairi Fauzi, Alwi Muhd Besari, Chan Yean Yean, Nabilah Ismail

**Affiliations:** 1 Microbiology Transfusion Unit, Department of Transfusion Medicine, Hospital Queen Elizabeth II, Lorong Bersatu Off Jalan Damai, Kota Kinabalu 88300, Sabah, Malaysia; 2 Bacteriology Unit, Infectious Disease Research Center (IDRC), Institute for Medical Research, National Institutes of Health (NIH) Complex, Setia Alam, Shah Alam 40170, Selangor, Malaysia; 3 School of Science, Monash University Malaysia, Bandar Sunway 47500, Selangor, Malaysia; 4 Department of Medical Microbiology & Parasitology, School of Medical Sciences, Universiti Sains Malaysia, Health Campus, Kubang Kerian 16150, Kelantan, Malaysia; 5 Hospital Universiti Sains Malaysia, Universiti Sains Malaysia, Health Campus, Kubang Kerian 16150, Kelantan, Malaysia; 6 Department of Emergency Medicine, School of Medical Sciences, Universiti Sains Malaysia, Health Campus, Kubang Kerian 16150, Kelantan, Malaysia; 7 Department of Medicine, School of Medical Sciences, Universiti Sains Malaysia, Health Campus, Kubang Kerian 16150, Kelantan, Malaysia

**Keywords:** Leptospirosis, febrile illness, pathogenic genes, genomic sequencing

## Abstract

Leptospirosis is an important worldwide tropical disease caused by pathogenic *Leptospira* spp. The determination of virulence genes is important, as it influences patients’ clinical manifestations and clinical outcomes. This case report focused on detecting the pathogenic genes of *Leptospira* in association with the clinical manifestations of patients at the Hospital Universiti Sains Malaysia, Malaysia, who presented with acute febrile illness. Two cases were found and, to the best of our knowledge, these were the first two cases in Malaysia in which patients presented with febrile illness were associated with successful *Leptospira* isolation from clinical samples. Both clinical isolates were identified by 16S rRNA sequencing as *Leptospira weilii* and *Leptospira interrogans*, respectively, and they were classified as pathogenic *Leptospira* by the presence of different pathogenic genes, based on a polymerase chain reaction (PCR) amplification of targeted genes. This report emphasizes that different infecting *Leptospira* species and the presence of different virulence factors cause a slight difference in clinical manifestations and laboratory findings of leptospirosis. Genomic sequencing and annotation revealed the detection of classical leptospiral virulence factor genes that were otherwise missed using PCR for detection of *Leptospira weilii* genome B208.

## 1. Introduction

Leptospirosis is recognized as a great mimicker because of its enormously wide variety of symptoms, ranging from subclinical diseases, such as a flu-like illness, to a severe syndrome of multi-organ infection with high mortality. The symptoms can imitate influenza, hepatitis, meningitis, viral hemorrhagic fever, and dengue fever. One study reported that 38% of the leptospirosis cases were misdiagnosed as hemorrhagic fever or dengue fever, due to similar clinical appearances [1]. The history of exposure and risk factors compatible with leptospirosis should alert clinicians to a possible diagnosis. Acute leptospirosis constantly presents with chills, headache, fever, conjunctival suffusion, vomiting, severe myalgia, nausea, anorexia, and malaise [2]. The vast majority of the infections are caused by pathogenic species, such as *L. interrogans, L. kirschneri, L. borgpetersenii, L. noguchii, L. santarosai, L. weilii,* and *L. alexanderi* [3]. The different species represent differences in DNA relatedness and possibly different geographical distributions, virulence, and clinical presentations.

The pathogenicity of *Leptospira* in humans are complex mechanisms that involve multi-protein interactions, including adhesion, that overcome host defense mechanisms followed by the expression of several virulence genes. Virulence genes are genes that code for factors, or for enzymes that produce factors, that are involved in interactions with the host; they are directly responsible for pathological damage during infection, and they are absent in nonpathogenic organisms [4]. 

This report focuses on detecting the pathogenic genes of *Leptospira* in association with the clinical manifestations of patients at the Hospital Universiti Sains Malaysia, Malaysia, who presented with acute febrile illness.

## 2. Description of the Cases

Here, we report two cases of leptospirosis patients in Malaysia who presented with febrile illness, in association with successful *Leptospira* isolation from clinical samples.

The first case (B208) was a 30-year-old man with no known medical illness who presented with febrile episodes for 3 days that were associated with myalgia, arthralgia, and headache. In addition, he had prominent gastrointestinal manifestations, presented with diarrhea for 5 days, and experienced poor oral intake. His further history revealed that he was involved with jungle trekking in a rural area, and that two of his companions developed similar symptoms. On admission to the medical ward, his vital signs were stable, with normal oxygenation and blood pressure. He was tachycardic and his body temperature was elevated. Physical examination was unremarkable, except for conjunctival suffusion. There was no hepatosplenomegaly and no palpable cervical lymphadenopathy. A laboratory investigation revealed slight leucocytosis at 12.5 × 10^9^/L (the normal range is 4 to 11 × 10^9^/L) and elevated C-reactive protein at >200 mg/L (the normal range is <10 mg/L), with otherwise normal blood cell counts. Liver and renal function tests were also normal. *Leptospira* IgM enzyme-linked immunosorbent assay (ELISA) and rapid *Leptospira* IgM Duo rapid test (ImmuneMed, Korea) were negative; however, an in-house real-time polymerase chain reaction (qPCR) was positive for *Leptospira* DNA and the isolation of *Leptospira* was also positive at day seven of cultivation. The patient was treated with intravenous ceftriaxone for 4 days, followed by 3 days of oral doxycycline, and he required intravenous hydration for 2 days. He subsequently became afebrile after 3 days of antimicrobial therapy and he was discharged 4 days after admission.

The second case (B004) was a 19-year-old man who was previously healthy and presented with a high-grade fever for 6 days. The febrile episodes were associated with nausea and persistent vomiting for 3 days, with epigastric pain and a poor appetite. On further questioning, the patient indicated that he had a history of swimming in a river about 10 days prior to the illness. As in the first case, the patient was tachycardic and had a raised body temperature on initial examination. Otherwise, the patient’s blood pressure and oxygenation were normal. On abdominal examination, there was palpable tender liver at a two-finger breadth below the costal margin. The rest of the physical examination was unremarkable, with no jaundice or conjunctival suffusion noted. A laboratory investigation revealed that the patient had leukocytosis, with otherwise normal blood counts. His C-reactive protein was elevated at more than 200 mg/L (the normal range is <10 mg/L). There was also renal involvement with urea at 10.6 mmol/L (the normal range is 2.5 to 6.7 mmol/L), and creatinine at 182 μmol/L (the normal range is 70 to 100 μmol/L). The liver function test was normal. The *Leptospira* IgM Duo rapid test result (ImmuneMed, Korea) was intermediate; however, the microscopic agglutination test (MAT) result was negative. Then, *Leptospira* DNA was detected by in-house qPCR, and the isolation of *Leptospira* was positive on day 11 of cultivation. The patient initially required intravenous fluid and was treated with parenteral ceftriaxone for 4 days; subsequently, this was stepped down to 3 days of oral doxycycline. The condition improved and he was discharged 4 days after admission, with advice for an outpatient review of his renal profile. 

## 3. Materials and Methods

Six ml of blood samples were collected from each patient prior to antibiotic administration and after obtaining their informed consent. The diagnosis of leptospirosis in both cases was confirmed by qPCR and positive isolation of *Leptospira* spp. Both clinical isolates, B208 and B004, were identified by 16S rRNA sequencing as *L. weilii* B208 (GenBank accession number JAMKEM000000000.1) and *L. interrogans* B004 (GenBank accession number JAMKEN000000000.1), respectively. Whole genomic sequencing for both isolates was performed and analyzed.

### 3.1. qPCR Detection of Leptospira

qPCR detection of *Leptospira* DNA was carried out, following a previous protocol [5]. Briefly, following DNA extraction, 8 µL of patient DNA was added to a PCR mix containing 1× Biorad SsoAdvanced™ Universal Probes Supermix, 200 nM forward and reverse primers, 100 nM probe, and PCR-grade water (adjusted to a total volume of 20 µL). The reactions were subjected to a thermal cycling condition, consisting of 95 °C (5 min) followed by 50 cycles of 95 °C for 30 s and 61.3 °C for 30 s.

### 3.2. Leptospira Isolation

The standard method to isolate *Leptospira* from the blood sample was by inoculating 1 to 5 drops (100 to 200 µL) of whole blood directly into EMJH media. The volume of the whole blood used for culturing was lower to avoid the inhibition of *Leptospira* growth by hemoglobin, antibiotics, antibodies, and other blood component factors [6,7]. The positive cultures from both patients were amplified and identified by PCR on the 16S rRNA gene by sequencing. In addition, the presence of the pathogenic genes was determined by using nine pathogenic genes: *lfb1, flaB, OmpL1, ligA, ligB, ligC, lipL21, lipL32*, and *lipL41*.

### 3.3. PCR Amplification of Virulence Genes

Amplification of the DNA was performed in a 25 µL reaction containing 1 mM of each primer, 12.5 µL of DreamTaq Green PCR Master Mix (Thermo Scientific, Malaysia), 2 µL of DNA template, and 8 µL of DNase-free water. The PCR cycling condition consisted of an initial denaturation step at 95 °C for 5 min, followed by 30 amplification cycles of denaturation at 95 °C for 30 s, annealing at a specified temperature for each primer for 30 s and extension at 72 °C for 30 s. A final extension step was performed at 72 °C for 5 min. The PCR cycles used in this study were based on the manufacturer’s recommendations for PCR Master Mix (Thermo Scientific, Selangor, Malaysia).

### 3.4. Genome Sequencing, Assembly, and Quality Control

Genomic DNA was extracted from bacterial isolates using an MN NucleoSpin Tissue Genomic DNA Purification Kit (Apical Scientific, Selangor, Malaysia). The genomic DNA was quantitated using a Multiskan Sky Microplate Spectrophotometer (Thermo Fisher Scientific, Waltham, MA, USA) and a Qubit Fluorometer (Bio-Diagnostics, Selangor, Malaysia) before being shipped for library preparation at Bio3 Scientific Sdn Bhd company (Selangor, Malaysia). DNA fragmentation was carried out using Covaris S220 (Covaris, Woburn, MA, USA), followed by end repair, dA-tailing, adapter ligation, and purification using a VAHTS Universal DNA Library Prep Kit for Illumina (Nanjing Vazyme Biotech Co., Nanjing, China). The Agilent 2100 (Agilent, Santa Clara, CA, USA) and the Qubit Fluorometer (Thermo Fisher Scientific, Waltham, MA, USA) were used to determine library quality. Whole-genome sequencing was performed on a Novaseq 6000 platform (Illumina, San Diego, CA, USA). Upon completion, sequencing reads were quality-filtered using a FastQC (http://www.bioinformatics.babraham.ac.uk/projects/fastqc/; access date 10 February 2022) and adapter and were trimmed off using TrimGalore (https://www.bioinformatics.babraham.ac.uk/projects/trim_galore/; access date 10 February 2022) and CutAdapt (http://code.google.com/p/cutadapt/; access date 10 February 2022) [8] before genome assembly using SPAdes (http://bioinf.spbau.ru/spades; access date 10 February 2022) [9]. The quality of the genome assembly was assessed via EvalG (https://patricbrc.org/app/Annotation; access date 10 February 2022) [10].

### 3.5. Genomic Annotation

Genome annotation was carried out using Bakta version 1.3.3 (https://github.com/oschwengers/bakta; access date 10 February 2022) (database schema 3) [11]. The protein database included the UniRef protein sequence cluster universe. Then, reported *Leptospira* virulence genes were searched from the genome annotations [12], according to UniRef protein IDs (Table 1):

Annotation of antimicrobial resistance genes and virulence factor genes was conducted using Abricate (https://github.com/tseemann/abricate; access date 10 February 2022) against the comprehensive antibiotic resistance database (CARD) database and the virulence factor database (VFDB) database, respectively [13,14].

### 3.6. Taxonomic Assignment and Phylogenomic Tree Construction

Taxonomic assignment was carried out using GTDBtk (https://github.com/Ecogenomics/GTDBTk; access date 10 February 2022) against GTDB database release 202. An ANI value of over 95% in GTDBtk confirmed the assignment of the B004 genome as *L. interrogans* and the B208 genome as *L. weilii*.

The two genomes were analyzed against closely related *Leptospira* genomes that were accessible from the NCBI GenBank and RefSeq databases, based on SNPs. SNP calls were made against the sample genome, using snippy (https://github.com/tseemann/snippy; access date 10 February 2022). For sample B004 (*L. interrogans*), another 31 *L. interrogans* were used together for phylogenomic tree construction (gls454012v02 assembly as outgroup). For genome B208 (*L. weilii*), another 18 *L. weilii* genomes were included, with assembly ASM200984v1 (*L. alexanderi* 56659) as the outgroup. Then, variant calls of respective *Leptospira* species were merged, using the snippy-core program.

The core SNP tree was constructed from merged variant calls using Gubbins v3.1.6 (https://github.com/nickjcroucher/gubbins; access date 10 February 2022) [15]. The phylogenetic tree was visualized using iTOL v6 (https://itol.embl.de/; access date 10 February 2022) [16].

### 3.7. Multi Locus Sequence Typing (MLST)

The MLST assignment was carried out against the pubMLST database, using the software MLST (https://github.com/tseemann/mlst; access date 10 February 2022) [17].

## 4. Results

### 4.1. Leptospirosis Investigation Results

The leptospirosis investigation results for both cases are summarized in Table 2.

### 4.2. Whole-Genome Sequencing

The taxonomy of *L. weilii* and *L. interrogans* was further confirmed based on whole-genome sequencing using GTDBtk (GTDB release 202). Both of the isolates were classified as pathogenic *Leptospira* and were determined by the presence of five and nine pathogenic genes, respectively, as shown in Table 3, based on the PCR amplification of the targeted genes. In this study, PCR products were amplified in all tested primers, suggesting that the second patient’s isolate expressed all nine of the tested pathogenic genes, while the first patient’s isolate only expressed *lfb1, flaB, ligB, ligC*, and *lipL32* genes.

MLST sequence type based on *Leptospira* scheme assigned genome B004 as sequence type 249, while there was no sequence type for genome B208. The raw output result from the MLST for *L. interrogans* and *L. weilii* are presented in Table 4 and Table 5, respectively.

### 4.3. Phylogenomic Study

**Figure 1:**
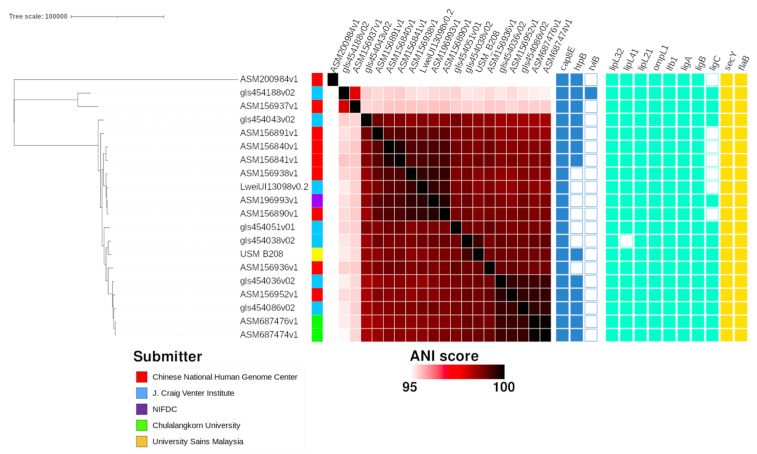
Phylogenomic tree of B208 genome against other L. weilii genomes. Metadata information provided in order are genome submitter, pairwise ANI score, predicted virulence genes in the virulence factor database (VFDB) and predicted virulence genes [12]. Assembly ASM200984v1 (*L. alexanderi*) acts as the outgroup.

**Figure 2:**
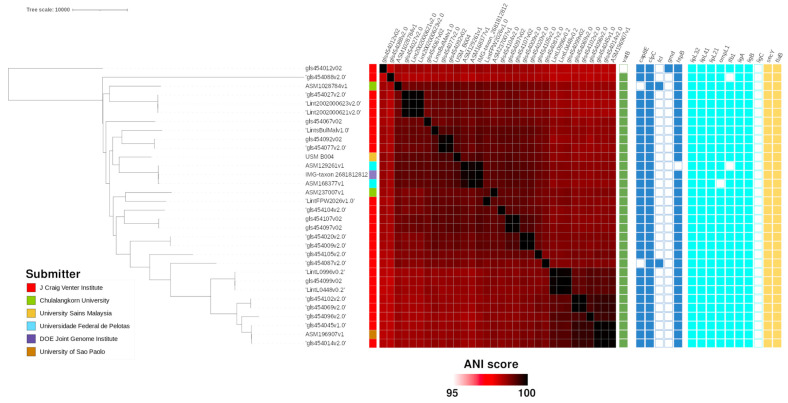
Phylogenomic tree of B004 genome against other L. interrogans genomes. Metadata information provided in order are genome submitter, pairwise ANI score, predicted antimicrobial resistance against the comprehensive antibiotic resistance database (CARD)and predicted virulence genes in VFDB, and predicted virulence genes [12].

The phylogenomic relationship of the B208 and B004 genomes conformed with the distribution of the virulence genes (Figure 1 and Figure 2).

## 5. Discussion

Leptospirosis is an endemic disease with global distribution, especially in Asia. In recent years, there have been increasing numbers of leptospirosis infection cases in Malaysia [18,19,20]. A case report showed that a real-time PCR assay was successfully used in a postmortem diagnosis of a woman whose death was caused by *L. interrogans* [21]. One study reported positively detected *Leptospira* cases and related risk factors in Sarawak, Malaysia [22]. Leptospirosis co-infected with other pathogens were normally seen in neighboring countries [23,24].

Advancements in molecular technology enable the expansion of the classical divisions of *L. interrogans* and *L. biflexa* into 64 species, based on DNA relatedness. These divisions are further classified into pathogenic species, non-pathogenic species, and species of indeterminate pathogenicity [25,26]. These classifications are quite different from the serologic classification, which may be of epidemiological value. The differences in genetic makeup among the pathogenic species may lead to the expression of different virulence factors, which may result in differences in clinical presentations [27].

Both of the clinical isolates in this study, *L. interrogans* and *L. weilii*, were pathogenic species. The slight difference in the presentations may be due to infection by different species of *Leptospira* with different virulence factors. *Leptospira interrogans* has a global distribution and there are some subgroups that are mostly isolated in the Asia–Pacific regions [28]. In comparison, *L. weilii* is a less commonly encountered species that has been previously reported in Australia [29]. In addition, the geographical distribution is related to the serovars [30]. However, a large number of patients infected by *Leptospira* have asymptomatic infections, particularly patients from endemic areas. Mild leptospirosis is the most common form of the disease, presenting in 90% of the cases [2] of patients who came from the endemic areas of leptospirosis.

Pathogenic *Leptospira* are responsible for human or animal infections. Although the pathogenic mechanisms of *Leptospira* are not clearly defined, potential virulence factors include lipopolysaccharide (LPS), OMPs, and adhesion molecule genes that are present in pathogenic *Leptospira* may help in understanding pathogenicity mechanisms. In animal cells, the pathways of Toll-like receptor 2 (TLR2) and Toll-like receptor 4 (TLR4) activate the host target, contrary to human cells that involve the activation of macrophage via TLR2 with the existence of CD14 [31]. The infection of *Leptospira* could result in problems with multiple organ systems or loss of life in unintentional hosts, such as humans, or merely moderate chronic or asymptomatic infections in reservoir hosts, including rodents [1]. All such primers were used to target the virulence factors and can be used to distinguish pathogenic from the saprophytic *Leptospira*. The differentiation of the pathogenic *Leptospira* is also crucial in classifying the pathogenic status for epidemiological and taxonomical study.

The role and contribution of individual virulence factors in the pathogenesis of leptospirosis are still not well defined. A combination of mechanisms, such as adhesions that allow adherence, immune-mediated responses, the ability of the host to recognize leptospiral LPS, toxin production, and surface proteins that result in immune evasion, may lead to a broad spectrum of clinical manifestation [32]. However, the spectrum and severity of clinical manifestations may also be influenced by several other factors, such as the duration of exposure to the pathogen, inoculum doses, and individual susceptibility.

Certain virulence factors found in pathogenic *Leptospira* can confer the ability to adhere to and enter mammalian host cells [12]. Several virulence factors, such as *lipL32* and leptospiral immunoglobulin-like genes *ligA, ligB*, and *ligC*, were only found in pathogenic *Leptospira* and not in their non-pathogenic counterparts [12,33]. The *lig* sequence is a virulence factor and plays a role in host cell attachment and invasion during *Leptospira* pathogenicity; *ligA* and *ligC* are present in a limited number of pathogenic serovar, while *ligB* is universally distributed among all of the pathogenic strains. Because *ligB* is present among all the pathogenic *Leptospira* strains, it may be useful in the identification and classification of *Leptospira* [34]. A drastic reduction in *L. interrogans* survival upon serum challenge was observed after experimenting with a concomitant and complete silencing of both LigA and LigB proteins by CRISPR-interference (CRISPRi) [35]. This process possibly signifies that *ligA* and *ligB* are virulence factors that enhance survival. In this study, we did not recover known *Leptospira* virulence factors from VFDB (Figure 1 and Figure 2). Based on the detection of known *Leptospira* virulence factors of known *L. interrogans* and *L. weilii* genomes from genome annotation, we found that many of those virulence factors were broadly shared across all analyzed genomes. Genomic detection of a complete set of targeted virulence factors in the *L. weilii* genome B208 (Figure 1) contradicted PCR detection, as amplification for genes *ligA, lipL21*, *lipL41*, and *ompL1* on genome B208 were negative (Table 3). This suggests that the PCR amplification method is limited to protein sequences of *L. interrogans*, while possibly failing for other *Leptospira* species. This highlights the importance of reassessing conventional leptospiral diagnostic methods. For instance, PCR methods will fail to detect genes whenever mutations occur at the primer binding sites, which will consequentially lead to failure of primers to hybridizes to the target gene. Future PCR detection methods should account for the dissimilarity in sequences found in other *Leptospira* virulence genes and target more conserved genomic regions that are exclusive to pathogenic *Leptospira* genomes.

Another virulence factor, the outer membrane protein (OMP), plays an important role in pathogen virulence mechanisms, because this protein may evade the host’s immune response [36]. OmpL1, LipL21, LipL32, and LipL41 have been used in this study to establish the pathogenicity of the isolate. All of the primers targeted a known gene sequence that was reported to be preserved among more than 200 of the pathogenic *Leptospira* serovars [34,36,37,38,39]. OmpL1 is a porin expressed in pathogenic *Leptospira* strains that allows the dispersion of hydrophilic solutes through the external membrane to the periplasm [40]. LipL32 is the most abundant protein in pathogenic *Leptospira*; it is absent in nonpathogenic organisms and expressed during human infection. It is mostly used in leptospirosis studies [36]. The sequence and expression of LipL32 are highly conserved among pathogenic *Leptospira* species. LipL41 is one of the immunogenic OMPs that is surface-exposed and it is expressed during infections [37,38]. In addition to those primers, FlaB and Lfb were used to identify pathogenic *Leptospira*. The FlaB primer only amplifies a specific fragment from pathogenic *Leptospira*. A previous study reported that the FlaB PCR-based approach is an effective method for distinguishing and identifying the pathogenic *Leptospira* isolates [41,42].

The intake of doxycycline effectively cured leptospiral infection in both cases. Our results showed that the gene vatB was present in the majority of our *L. interrogans* genomes, but it was absent in *L. weillii* genomes, which suggest its role in *L. interrogans* survival, unlike its role with respect to *L. weillii.* If vatB is of no importance, the expression of vatB presents as a metabolic liability to *L. interrogans*. This indicates that, despite observations of horizontal gene transfer (HGT) in other pathogens, such as *Acinetobacter baumannii* [43], *Staphylococcus aureus* [44], and *Vibrio cholerae* [45], the conservation of the antimicrobial resistance (AMR) profile across *Leptospira* genomes suggests that HGT occurs minimally in *Leptospira*.

## 6. Conclusions

This report emphasized that different infecting *Leptospira* species and the presence of different virulence factors cause a slight difference in clinical manifestations and laboratory findings of leptospirosis. Genomic sequencing and annotation revealed the detection of classical leptospiral virulence factor genes that were otherwise missed using PCR for detecting *L. weilii* genome B208. Further large-scale investigation is needed to study the broad clinical manifestation of the disease in relation to species or serovar variation, antimicrobial susceptibility testing, virulence, and/or the pathogenicity of the *Leptospira* species.

## Figures and Tables

**Table 1:** Detection of *Leptospira* virulence factor genes by Bakta version 1.3.3.

Gene	IDs	Note
*lipL32*	UniRef90_Q6J0P4, UniRef50_Q6J0P4	In *L. interrogans* genomes, the last three genomes have different versions In *L. weilii* genomes, UniRef90_Q6J0P4 annotated as spirochaetales surface lipoprotein
*lipL41*	UniRef90_A0A2M9XPR1, UniRef100_X5FKY1, UniRef90_A0A1D7V0C6	
*lipL21*	UniRef90_Q04WF0	
*ompL1*	UniRef90_Q6GXE0	
*lfb1*	UniRef90_E7DSE3, UniRef90_E7DSD4	All *L. weilii* has only UniRef90_E7DSD4 form
*ligA*	UniRef90_Q72MA6, UniRef90_Q8EYU4	
*ligB*	UniRef90_A0A540TD47, UniRef90_Q04UY1	All *L. weilii* has only UniRef90_Q04UY1 form
*ligC*	UniRef_C0J1R0	
*secY*	UniRef90_Q9XD16, UniRef90_M3CP76	Housekeeping gene
*flaB*	UniRef90_O51941	Housekeeping gene

**Table 2:** Summary of leptospirosis investigation results.

Laboratory Test	Patient 1 (B208)	Patient 2 (B004)	Manufacturer
*Leptospira* IgM ELISA	Negative	-	Panbio, US
*Leptospira* IgM Duo Rapid	Intermediate	Negative	ImmuneMed, Korea
Microscopic agglutination test (MAT)	Negative	-	In-house
*Leptospira* in-house PCR	Positive	Positive	In-house
culture	Positive	Positive	In-house
16S rRNA sequencing	*Leptospira weilii*	*Leptospira interrogans*	Apical, Malaysia
Genomic characteristics:			
Chromosome size (bp)	4,298,595	4,858,647	
Number of contigs	220	169	
N50	106460	83016	
GC content (%)	40.72	35.09	
No. of coding sequences	3854	3884	
No. of RNAs rRNA	3	3	
tRNA	36	37	
tmRNA	1	1	
ncRNA	4	4	
CRISPR	1	3	
GenBank accession no.	JAMKEM000000000.1	JAMKEN000000000.1	

**Table 3:** Detection of pathogenic genes of the isolates from positive cultures, based on PCR amplification.

Pathogenic Gene	Target Gene	Patient 1 (B208)	Patient 2 (B004)
*ligA*	*ligA*	-	+
*ligB*	*ligB*	+	+
*ligC*	*ligC*	+	+
*lipL21*	*lipL21*	-	+
*lipL32*	*lipL32*	+	+
*lipL41*	*lipL41*	-	+
*flaB*	*flaB*	+	+
*lfb1*	*lfb1*	+	+
*ompL1*	*ompL1*	-	+

**Table 4:** Raw output result from the MLST software for *L. interrogans*.

Genome	Scheme	ST	glmU_1	pntA_1	sucA_1	tpiA_1	pfkB_1	mreA_1	caiB_1
gls454012v02	*Leptospira*	61	11	7	2	18	27	8	3
gls454088v2.0	*Leptospira*	252	1	10	7	8	15	5	52
ASM1028784v1	*Leptospira*	50	6	8	2	2	9	7	5
gls454027v2.0	*Leptospira*	51	6	13	2	2	13	2	6
Lint2002000621v2.0	*Leptospira*	51	6	13	2	2	13	2	6
Lint2002000623v2.0	*Leptospira*	51	6	13	2	2	13	2	6
gls454067v02	*Leptospira*	24	1	4	2	1	5	3	4
LintsBulMalv1.0	*Leptospira*	112	1	1	1	2	2	1	2
gls454077v2.0	*Leptospira*	49	5	1	1	1	3	2	7
gls454092v02	*Leptospira*	49	5	1	1	1	3	2	7
USM_B004	*Leptospira*	249	1	1	9	2	6	3	6
ASM129261v1	*Leptospira*	-	1	1	2	2,2	-	4	8
ASM168377v1	*Leptospira*	-	1	1	2	65	29	4	-
IMG-taxon_2681812812_ annotated_assembly	*Leptospira*	17	1	1	2	2	10	4	8
LintFPW2026v1.0	*Leptospira*	47	4	18	2	2	3	3	5
ASM237007v1	*Leptospira*	33	1	18	1	4	4	5	3
gls454104v2.0	*Leptospira*	83	1	1	1	27	6	5	2
gls454097v02	*Leptospira*	77	1	3	1	25	6	6	2
gls454107v02	*Leptospira*	86	1	18	1	25	6	6	2
gls454009v2.0	*Leptospira*	111	20	1	1	4	6	6	5
gls454020v2.0	*Leptospira*	111	20	1	1	4	6	6	5
gls454105v2.0	*Leptospira*	84	1	2	2	29	4	5	9
gls454087v2.0	*Leptospira*	42	3	11	3	2	4	5	8
LintL0996v0.2	*Leptospira*	46	4	1	1	4	4	6	6
LintL0448v0.2	*Leptospira*	46	4	1	1	4	4	6	6
gls454099v02	*Leptospira*	46	4	1	1	4	4	6	6
gls454069v2.0	*Leptospira*	37	3	3	3	3	4	5	5
gls454102v2.0	*Leptospira*	37	3	3	3	3	4	5	5
gls454096v2.0	*Leptospira*	80	3	1	17	3	4	5	16
gls454045v1.0	*Leptospira*	140	3	3	3	3	4	5	16
gls454014v2.0	*Leptospira*	140	3	3	3	3	4	5	16
ASM196907v1	*Leptospira*	140	3	3	3	3	4	5	16

**Table 5:** Raw output result from the MLST software for *L. weilii*.

Genome	Scheme	ST	glmU_1	pntA_1	sucA_1	tpiA_1	pfkB_1	mreA_1	caiB_1	Order
ASM200984v1	*Leptospira*	207	53	67	63	59	73	57	53	19
gls454188v02	*Leptospira*	192	47	57	60	52	61	50	46	18
ASM156937v1	*Leptospira*	191	50	62	58	54	65	53	48	17
gls454043v02	*Leptospira*	-	48	63	59	51	69	52	45	16
ASM156891v1	*Leptospira*	194	49	60	55	51	69	52	45	15
ASM156840v1	*Leptospira*	-	48	63	59	53	69	49	45	14
ASM156841v1	*Leptospira*	-	48	63	59	53	60	49	45	13
ASM156938v1	*Leptospira*	-	49	63	55	51	69	49	45	12
LweiUI13098v0.2	*Leptospira*	190	49	63	59	51	60	49	45	11
ASM196993v1	*Leptospira*	-	49	63	55	51	80	49	45	10
ASM156890v1	*Leptospira*	182	46	56	55	51	60	49	45	9
gls454051v01	*Leptospira*	-	48	63	59	51	69	54	45	8
gls454038v02	*Leptospira*	-	49	61	57	51	64	49	45	7
USM_B208	*Leptospira*	-	49	61	57	53	63	52	45	6
ASM156936v1	*Leptospira*	-	48	58	59	51	63	49	47	5
gls454036v02	*Leptospira*	-	49	59	57	53	63	49	45	4
ASM156952v1	*Leptospira*	-	46	59	57	53	63	55	47	3
gls454086v02	*Leptospira*	183	46	59	57	53	63	49	45	2
ASM687476v1	*Leptospira*	94	46	59	57	53	63	52	45	1
ASM687474v1	*Leptospira*	94	46	59	57	53	63	52	45	0

## Data Availability

The data presented in this study are available on request from the corresponding author.
